# The Self-Concept and Its Relationship with Parental Socialization and Environment in Primary School Students

**DOI:** 10.3390/bs14070518

**Published:** 2024-06-21

**Authors:** Antonio Sánchez-Urrea, Tomás Izquierdo-Rus, Salvador Baena-Morales, Alberto Gómez-Mármol

**Affiliations:** 1 Department of Plastic, Musical and Dynamic Expression, Faculty of Education, University of Murcia, 30100 Murcia, Spain; antonio.sanchez24@um.es (A.S.-U.); alberto.gomez1@um.es (A.G.-M.); 2 Department of Methods Research and Diagnosis in Education, Faculty of Education, University of Murcia, 30100 Murcia, Spain; 3 Department of General Didactics and Specific Didactics, Faculty of Education, University of Alicante, 03690 Alicante, Spain; salvador.baena@ua.es

**Keywords:** self-concept, parental socialization styles, socio-economic level, health, education

## Abstract

Self-concept in this article is considered in physical education, with the physical self-concept being in the foreground, and it is analyzed in this quantitative study to determine whether it is related with socio-economic environment and parental styles, focusing on primary education students in the Region of Murcia. Primary education students are in a critical period for the development of self-concept. To assess self-concept levels, socio-economic and cultural environments as well as parental socialization styles, the PSPP, NSE and ESPA29 questionnaires were respectively used. They were used to collect data from 937 students across various educational centers in upper courses (4th, 5th and 6th grades of primary education) to uncover realities in child society and their relationship with self-concept. In the first objective, parental socialization styles are shown to be related with self-concept. However, in the second objective, it is seen that the socio-economic environment is more related with self-concept than parental styles. Finally, the results of the third objective show that there is indeed a relationship with the socio-economic environment and the student body self-concept, both at the family level and for fathers and mothers independently. It is important to consider both the parental styles and the socio-economic environment in which primary education students develop for the development of self-concept. Additionally, educational implications and new lines of research are suggested in this topic.

## 1. Introduction

Families play a fundamental role in the development of self-concept and the health of primary education students. Tripon and Gabureanu [1] pointed out that the way in which each parent relates to his or her own child is connected with the education they receive as an adult, their educational level, their values, their attitudes and their conception of their world and life. The parenting styles experienced by students play a crucial role in the comprehensive development of their personality [2]. González-García [3] consider that students who receive love, sensitivity and a consistent upbringing are more likely to experience favorable development. The OECD [4] considered that the capacity of educational settings to support family engagement is also closely linked with children’s learning and well-being.

On the other hand, families in unfavorable situations (low socio-economic and socio-cultural levels) are associated with a higher risk of psychopathology in children and adolescents [3]. Lolín, Cabrera and Venegas [5] describe parental socialization styles as follows. The democratic style seeks to exert firm control over the behaviors of primary school students in a rational manner with a problem-oriented approach. This style encourages open communication by providing reasons for household rules and listening to the child’s opinions on these matters. The permissive parental style is characterized by parents managing their child’s impulses and actions without punishment, accepting and affirming their child’s personality. In contrast, authoritarian parents aim to model and evaluate their child’s behavior and attitudes according to socially desirable conduct standards, exercising high levels of control and demanding a high degree of maturity. Finally, Rollins and Thomas [6] consider that a neglectful parental style demonstrates low levels of involvement and demand, with a lack of structure, control, support and engagement in the child’s behaviors. Such parents tend to delegate their parental responsibilities to other figures, such as the school or other family members [7].

Huffman et al. [8] found that permissive families have children with a better development of self-concept compared to children from authoritarian families. However, the same authors also linked students from permissive families in physical education with less healthy behaviors. In addition, it has been observed that democratic families are associated with higher levels of self-concept [9]. In the field of physical education, Pelegrín et al. [2] observed that children from authoritarian families tend to show lower levels of interest in class compared to children from permissive families. It is essential to understand that various aspects related to the family modulate the level of self-concept of primary education students. The parental style that prevails in the student’s home can affect their lifestyle, particularly in their sports and eating habits, as has been gathered from the studies analyzed. This can lead to a lack of healthy habits and lifestyles, since what students value, acquire and apply during their developmental period will have a decisive impact on their future quality of life, whether healthy or not. In summary, parents have an important role in the development of their children. Consistent upbringing and love are fundamental for the emotional and physical well-being of students. Democratic and permissive parenting styles are associated with higher levels of self-concept, while authoritarian styles can negatively affect students’ interest in physical education. It is important for parents to foster healthy habits and lifestyles in their children from an early age.

### Self-Concept and Socio-Economic and Cultural Environments

Self-concept is defined as the perceptions a person holds about themselves. These perceptions are shaped through personal experiences and interpretations of the environment and are particularly modulated by the reinforcements and evaluations of significant others, as well as by self-attributions regarding one’s own behavior [10]. Self-concept is the perception we have of ourselves and is influenced by external factors such as parenting styles and socio-economic and cultural environments [9]. Various studies have shown that self-concept is a determining factor for success in academic performance, social relationships, and participation in physical and sports activities [11]. Marsh and Shavelson [12] classify self-concept as follows: (1) multidimensional or multifaceted; (2) hierarchical; (3) stable when referring to general self-concept, and increasingly unstable as one moves down the hierarchy; (4) with multidimensionality becoming more evident as an individual progresses from childhood to adolescence; (5) containing two types of dimensions, evaluative and descriptive; (6) distinguishable from other constructs such as academic achievement.

According to Torres-Mejía et al. [13], in the case of primary education children, it has been observed that those who have a healthy and active routine possess a good self-concept. On this point, with regard to age, it is important to remark that older grades (adolescents in secondary education) are in a period of a higher search of a self-identity in comparison to primary education students, who participated in this study. Furthermore, body image improves self-concept and favors mood [14]. Arrayás et al. [15] consider that during adolescence, women experience greater body dissatisfaction than men, which can lead to avoiding social situations where their bodies are exposed. Teachers play a fundamental role in promoting a supportive and positive valuation environment that contributes to strengthening their students’ self-concept, thus promoting their emotional and social well-being. For this, it is important to carry out a prior analysis of the students’ self-concept and to guide the teaching–learning process appropriately. In conclusion, self-concept is a fundamental aspect in the personal development of students, and teachers should consider the factors that affect its formation and evolution to foster a positive self-concept in young people.

The relationship between cultural and socio-economic environments and the development and health of primary education students is a topic of great importance. According to Wassenaar et al. [16], developed countries, where most of the research in this area has been conducted, have different indices of socio-economic wealth and inequality compared to poorer countries and represent a minimal proportion of the world’s children. A growing body of research points to associations between cognitive performance and lifestyle, such as physical activity, sedentariness, obesity, healthy eating and socio-economic and cultural status, among others [17]. Joaristi et al. [18] showed how students from low socio-economic backgrounds face greater difficulties in accessing an active and healthy lifestyle, while students from high socio-economic backgrounds are more likely to possess a better-formed state of self-concept. Poverty, a vulnerable social environment, and low socio-economic status have a significant impact on brain and body health [16]. This socio-economic disparity affects students unequally, reflecting in their health and well-being. Teachers must recognize these factors to understand the reality of their students and design appropriate educational strategies that promote the adoption of healthy lifestyles and the improvement of self-concept in this context. From the perspective of Izquierdo-Rus et al. [19], two groups were identified that influence the healthy development of primary education students: firstly, the climate and family functioning at home, including the time family members dedicate to the children, involvement in their upbringing as well as the lifestyles, norms and attitudes established in the family environment; secondly, the economic level of families, their labor status, economic situation and academic level and the cultural resources available to the children. According to Colombo [20], although in Western economies generally it is common practice to reach a third-level degree, in the Region of Murcia the school dropout rate, especially at secondary school level, is high and closely linked to the socio-economic context, which involves aspects of culture. In summary, both cultural and socio-economic environments have a significant impact on the health status and development of primary education students. It is important for teachers to take these factors into account in designing appropriate educational strategies that promote the adoption of healthy lifestyles and the improvement of self-concept in this context. Additionally, measures should be taken to promote healthy cultural and socio-economic environments in schools.

This research addresses the intersection between parental education, socio-economic and cultural environments and the self-perception of primary education students, identifying potential gaps in current research. Despite significant advancements in this field, there are still areas requiring deeper exploration, particularly in understanding how these factors interact and impact the development of self-concept in young people and adolescents. Therefore, this study focuses on three main objectives: Firstly, to analyze the relationship between parental socialization styles and self-concept in the field of physical education (Objective 1), positing that the parental style received significantly affects the self-concept of primary education students (H1). Secondly, to determine the levels of self-concept modulated by parental socialization styles and socio-economic and cultural environments (Objective 2), hypothesizing that the development of self-concept is affected by both parental styles and socio-economic and cultural contexts (H2). Thirdly, to explore whether there is an association between socio-economic environment and self-concept in primary education students (Objective 3), under the hypothesis that socio-economic environment plays a crucial role in shaping students’ self-concept (H3). In this way, the study aims to address the complexity of factors based on young people’s development comprehensively and thoroughly, thereby contributing to a deeper understanding and the formulation of effective strategies to support their holistic growth.

## 2. Materials and Methods

### 2.1. Approach and Design

To describe the approach and design of this research, we draw on the considerations of Hernández-Pina et al. [21], who explained the steps followed in the development of the research. According to these authors, the information provided is sufficiently comprehensive and detailed so that other researchers can use it in a different context. In this research, a positivist and quantitative approach is adopted, as suggested by Thomas and Nelson [22]. This implies the use of quantitative techniques to diagnose and resolve a problem. The research design is classified as non-experimental, meaning that phenomena that have already occurred are studied, and variables cannot be controlled. It is an ex post facto design, as mentioned by Bisquerra [23]. The main objective of the research is to describe the current state of certain variables and explore possible correlations or associations between them. The study comprises non-experimental quantitative research, whose purpose is to analyze and assess existing conditions as well as examine the relationships between different aspects without directly manipulating the variables [24]. It is highlighted that this research is based on a survey, which is a data collection technique where a sample of participants is selected and questionnaires are applied to them [25]. These questionnaires contain questions that provide the necessary information to address the research problem [25].

Bevins [25] pointed out that the objective of ex post facto studies is to validate or refute research hypotheses once a phenomenon has occurred. In other words, this type of study involves a “retrospective” search for the possible causes that have led to the phenomenon. In this case, parental socialization styles and physical activity are investigated.

### 2.2. Participants and Context

The present study involved 937 students, aged between 9 and 14 years, attending the fourth, fifth and sixth grades of primary education. The sample selection was carried out in 12 schools, of which 7 were public and the remaining 5 were privately owned or subsidized. Among these students, we found a majority with a biparental family structure. They were mostly of Spanish origin, but in the different areas where the data were collected, there is cultural diversity, with the presence of Moroccan and gypsy communities. These students spend an average of 6 h in school, with the remaining time spent either in extracurricular activities or with their families.

We chose the Region of Murcia as the study area, where the economy is significantly based on agriculture, making it one of Spain’s leading producers of fruits and vegetables, which in turn drives the agro-food industry. However, this dependence also makes the economy vulnerable to climate change and price fluctuations in agricultural products. The region has faced higher-than-average unemployment rates, with a large proportion of the workforce engaged in temporary and seasonal jobs in agriculture and tourism, leading to economic instability for many workers. Additionally, there are significant disparities in income and living conditions within the region, with rural areas and certain urban neighborhoods experiencing higher levels of poverty and social exclusion, reflected in limited access to education, healthcare and other essential services. Following the considerations of Hernández-Pina et al. [21], the sample was census-based, and participants were invited to be part of the research study on a voluntary basis. Table 1 presents the sociodemographic characteristics of the participants for observation.

A data cleaning process was carried out to verify and eliminate cases that presented missing values related to the variables of interest. However, no cases with missing values that could affect the sample size were identified. Following this presentation of the characteristics of the participants and the context, the next section details the data collection instruments.

### 2.3. Data Collection Instruments

A specific questionnaire was used to assess each of the research variables. For parental socialization styles, the Parental Socialization Scale (ESPA29) was used, which has been validated by Musitu and García [26]. This scale consists of 29 items grouped into two factors, communication (e.g., “if I respect the home rules related to times…”) and control (e.g., “if I have a good behavior at home…”), addressing situations of obedience and disobedience. The used scale is of the Likert type, with four response options ranging from “never” to “always” to measure the categories of perceived parental socialization styles: permissive, authoritarian, democratic and negligent. It is worth mentioning that this questionnaire has been previously used in research conducted by Martínez et al. [27], García Ponce and Gómez-Mármol [28] and Huamán-Chura [29]. Additionally, Deeba, Saleem and Ulla [30] used a Likert scale method to find students’ extent of academic self-concept to further correlate it with their obtained marks in English.

To assess physical self-concept, the Physical Self-Perception Profile (PSPP) questionnaire was used, developed and validated by Fox and Corbin [31], in its Spanish version translated by Moreno and Cervelló [32]. This questionnaire consists of 30 items, grouped into five factors: physical condition (e.g., “I always keep an excellent physical condition”), appearance (e.g., “I am always satisfied with my physical appearance”), perceived competence (e.g., “I am very good at almost any sport”), strength (e.g., “My muscles are as strong as the rest people’s muscles of my own sex”), and self-esteem (e.g., “I believe I am between the most skilled partners when a sport ability is involved”). Each factor is composed of different statements related to physical self-concept, and the responses are collected on a Likert scale with four options, ranging from “Strongly disagree” to “Strongly agree”. The scale includes an introductory phrase that states “When I do physical activity…”. It is important to highlight that this questionnaire has been used in both international and national research, as in the studies by Yang and Wu [33], Ruiz-Montero et al. [34] and Christiansen et al. [35]. Internal consistency values were calculated using Cronbach’s alpha coefficient, with the following results: physical condition α = 0.722, appearance α = 0.740, perceived competence α = 0.781, strength α = 0.728 and self-esteem α = 0.669.

To gather information about the socio-economic and cultural environments, a socio-economic and cultural level (NSE) measurement instrument was used, validated by Joaristi et al. [18]. In this case, the focus was specifically on the first block of the instrument, which consists of 4 items related to the family unit. These items address the number of people living in the home, nationality, the education level of the parents and employment status. Based on this information, subjects were classified into three socio-economic levels, high, intermediate and low, using the categorization criteria of Goldthorpe [36]. It is relevant to mention that this instrument has also been used in previous research, such as that conducted by Srivastava and Bhatia [37] and Pérez et al. [38].

### 2.4. Procedure

The various stages carried out during the development of the research are described. The first phase involved establishing the research questions, objectives and study design. Then, ethical approval was requested from the corresponding committee of the University of Murcia for the use of the questionnaires that would be employed in the research, which already had such prior approval. Once the approval of the questionnaires was validated, an email communication was sent to all of the primary education centers in the Region of Murcia to inform them about the study’s theme.

Once authorization from the educational centers to participate in the study was obtained, a more detailed informed consent document was sent describing the study’s information, including the questionnaires to be used and the subsequent analysis of the collected data. An informed consent document was also provided to the families to ensure the full participation of the students. The next phase of the procedure consisted of administering the questionnaires by the teachers of each course, with the collaboration, in some cases, of the researcher. The questionnaires were completed in printed format during school hours in an appropriate environment that ensured privacy and concentration without any external pressure and respecting the anonymity of the participants. It was ensured that all items of the questionnaire were answered, and the duration of administration ranged between 40 and 60 min.

### 2.5. Data Analysis

The data treatment was carried out using the statistical software SPSS 24.0. Additionally, an analysis of the data distribution was carried out using Kolmogorov–Smirnov tests and χ^2^ for quantitative and qualitative variables, respectively. To determine the relationship between variables, Kruskal–Wallis H tests, Pearson’s chi-square and linear regression were used.

## 3. Results

The analysis of the results is presented in relation to the objectives and hypotheses posed. Next, the first objective of the research is analyzed—that is, studying the connection between parental socialization styles and self-concept in the field of physical education. The association between parental socialization styles and the self-concept of primary education students is shown, and for this analysis, the Kruskal–Wallis H formula is used (Table 2).

The data presented in the aforementioned table reveal that within children’s self-concept, satisfaction with physical condition is the highest, while satisfaction with strength is the lowest in the entire sample. In relation to parental socialization styles, significant differences are observed in all dimensions of self-concept. The democratic parental socialization style shows the lowest level of self-esteem, while the authoritarian parental style presents the lowest levels of satisfaction with physical appearance, physical condition and strength. Moreover, it is highlighted that the permissive parental socialization style has the highest values in the subcategories of self-concept, while the negligent parental style shows the lowest level of satisfaction in perceived competence. Next, the analysis of the second objective is presented to determine the levels of self-concept mediated by parental socialization styles and socio-economic and cultural environments. Table 3 shows the results obtained with the linear regression formula.

In Table 3, it can be observed how self-concept is modulated, in all its categories, by the socio-economic environment. Physical condition and self-esteem show values closer to 0.0, and strength is closer to 0.5. However, in this relationship, it can be contemplated how parental socialization styles do not show a significant relationship with self-concept, contrary to what was seen in the first objective. Lastly, the analysis of the third objective is presented to determine the levels of self-concept with regard to socio-economic environment. Table 4 shows the results obtained with the Kruskal–Wallis formula.

Referring to Table 4, it can be seen how self-concept is mediated in most categories by the family socio-economic environment in which the students live. It is observed that strength is the only category that does not have a significant relationship with the family socio-economic environment, while the rest of the categories do show a significant association. Additionally, these results prompt an interest in understanding what happens when self-concept is associated with the socio-economic level of the mother and father separately. In Table 5, we see the results of the analysis between self-concept and the mother’s socio-economic level, which was conducted using the Kruskal–Wallis test.

From Table 5, it can be deduced that perceived competence is the one category that does not have a significant relationship with the mother’s socio-economic level, while the rest of the categories do. Next, in Table 6, self-concept is analyzed alongside the father’s socio-economic level to determine whether there is a significant difference or not. For these analyses, the Kruskal–Wallis test is used again.

After analyzing Table 6, it can be observed that strength is the only category that does not have a significant difference with the father’s socio-economic level, while the remaining categories do. These results are related to those in Table 3, where it is also observed that strength does not show an association with the family socio-economic level.

## 4. Discussion

The findings pertinent to the primary objective of this research, which aimed to analyze the impact of parental socialization styles on self-concept within the context of physical education, unveil two critical insights. Firstly, the study illuminates the significant role of parental socialization styles in shaping the self-concept of participants. This connection underscores the intricate link between family dynamics and personal development in young learners. Secondly, the research highlights the distinct effects of varying parental socialization styles, illustrating how they differentially affect the constituent categories of self-concept. These findings suggest that the nature of parental interaction, whether permissive, authoritarian, democratic or negligent, plays a pivotal role in the development of key aspects of self-concept among primary education students.

In this sense, Villarejo et al. [39], in a study focused on adolescents and examining the influence of parental socialization styles on self-esteem, found that the permissive style has a greater impact on development compared to the authoritarian style. However, no correlation was found between the participants of Villarejo et al. [39] and the primary education students in this research. At the European level, studies such as those by Calafat et al. [40] and García et al. [41] have determined that the permissive style, characterized by acceptance without imposition, is considered the most suitable for the development of self-esteem in primary education students. This research adds that the permissive style has a stronger relationship with all categories of self-concept in primary education students. On the other hand, the subjects in this research demonstrated that the authoritarian style has the lowest correlation with physical appearance, physical condition and strength. Furthermore, it was observed that the democratic style has a low connection with the development of self-esteem, and the negligent style barely affects perceived competence, which constitutes one of the categories of self-concept. In a study conducted in Latin America, Martínez et al. [42] demonstrated that the permissive style has a stronger relationship with the development of self-esteem as part of the self-concept construct. These findings are in line with the results of the present research, where it was observed that the permissive style is the most determinative in the development of the self-concept of primary education students. On the other hand, research conducted in Anglo-Saxon countries, such as that by Aunola et al. [43], Baumrind [44], Darling and Steinberg [45] as well as Maccoby and Martín [46], points to the authoritarian parental style as a principal factor in the development of self-concept. However, in contrast to these studies, the present research revealed that the authoritarian parental style has the weakest relationship with the development of physical appearance, physical condition and strength. Additionally, it is noted that the negligent and democratic styles are linked with lower levels of self-esteem and perceived competence, while the permissive style reports the highest levels in all categories of self-concept. Gómez-Ortiz et al. [47] and Gómez-Ortiz et al. [48] pointed out the importance of parental socialization styles as determinants, either positively or negatively, in various aspects of the psychosocial development of primary education students. These findings align with the results obtained in this research, confirming the connection between parental socialization styles and students’ self-concept. Domènech [49] concluded that permissive and negligent parental styles correlate with the development of self-concept in primary education students. However, in this doctoral study, it was observed that it is the permissive parental style that has a greater relationship with the various factors of self-concept. Moreover, Pérez-Gramaje [50] emphasized that parental socialization styles are the first processes of socialization in a child’s life and play a fundamental role in the formation of identity and the internalization of social norms. These socialization processes begin from birth and intensify during adolescence [50,51]. Based on the results obtained in the first specific objective, where the statistically significant relationship between educational styles and students’ self-concept was analyzed, the significant association between parental socialization styles and students’ self-concept is confirmed.

Regarding the second objective, the present research sought to examine the connection of parental styles with socio-economic environment in relation to the self-concept of adolescents. The results indicated that the socio-economic environment has a significant impact on self-concept, supporting previous studies that have found an association between these factors [52,53]. These findings align with earlier research that has demonstrated the influence of the socio-economic environment on the formation of adolescents’ self-concept. For instance, Smith et al. [53] examined the relationship between socio-economic status and self-concept in a sample of adolescents from various backgrounds. They found that those from more favorable socio-economic environments had a more positive self-concept compared to those from disadvantaged socio-economic environments. These findings align with the results of our study, supporting the idea that the socio-economic contexts in which adolescents grow up can condition their self-perception. Additionally, Van Dommelen et al. [54] investigated the connection between socio-economic status and the self-concept of adolescents in a sample from different countries. They found that adolescents from higher socio-economic backgrounds tended to have a more positive self-concept compared to those from lower socio-economic backgrounds. These results support our research, suggesting that the socio-economic environment plays a significant role in the development of self-concept. However, surprisingly, our results did not show a significant difference in self-concept in relation to parental styles. This contrasts with previous studies that have highlighted the importance of parental styles in the development of adolescents’ self-concept [55]. These studies have found that parental styles, such as authority, emotional support and open communication, change how adolescents view themselves and their self-esteem. For example, Pérez-Gramaje [52] examined the relationship between parental styles and self-concept in a sample of Spanish adolescents. He found that parental styles characterized by greater emotional support and open communication were associated with a more positive self-concept in adolescents. These findings highlight the importance of parental styles in the formation of self-concept. Similarly, Sabeh [55] investigated the relationship between parental styles and self-concept in a sample of Lebanese adolescents. His results showed that authoritarian parental styles were associated with a more negative self-concept in adolescents, while democratic parental styles were related to a more positive self-concept. These results support the notion that parental styles play a crucial role in the development of adolescents’ self-concept.

Given these unexpected results in our study, it is important to consider that other factors not considered here might be affecting the relationship between parental styles and self-concept. For example, future research could examine the family relationship quality, social peer pressure and other aspects of the social environment with regard to the development of adolescents’ self-concept [48,56]. These factors could contribute to a more complete understanding of how self-concept is shaped in relation to parental styles. In conclusion, while our study found a significant connection between the socio-economic environment and the self-concept of adolescents, no significant difference was found in relation to parental styles. These unexpected results contrast with previous studies that have emphasized the importance of parental styles in the development of self-concept. However, it is important to remark that other factors not explored in this study could be affecting this relationship. Parental education and socio-economic status are significant determinants of children’s self-concept, but they are not the only factors. Consequently, it would be beneficial to develop future research that assesses the role of school factors, such as social comparison processes, in the genesis of self-concept in follow-up studies.

In the third objective of this study, the association between the socio-economic environment and the self-concept of primary education students was examined. The results indicated that there is a significant relationship between the socio-economic environment and self-concept in most of its categories, except for strength, suggesting that factors related to socio-economic level can affect students’ perception of themselves. These findings are in line with previous research that has demonstrated a relationship between socio-economic environment and self-concept. For example, the meta-analysis conducted by Sirin [57] found a significant association between socio-economic status and students’ academic performance. This supports the idea that socio-economic conditions can impact the way individuals view themselves. Furthermore, the study by Leung et al. [58] revealed that low-income adolescents exhibited higher levels of aggression and internalized problems compared to their higher socio-economic status counterparts. These results suggest that socio-economic disparities can condition how young people perceive and experience different aspects of their self-concept, thus being related to the findings of this research, where the socio-economic environment was found to have a direct relationship with self-concept. In the family context, Leung et al. [58] found that the economic well-being of families was related to children’s social adjustment, supporting the notion that the socio-economic environment can affect the development of their self-concept. Additionally, Marín [59] highlighted the importance of considering socio-economic status when examining child development, including self-concept.

Therefore, the results of this study support previous research that has found a significant association between the socio-economic environment and self-concept. These findings underscore the importance of considering socio-economic context when analyzing the development of self-concept in students. By comparing the results of this study with previous research, the evidence of the connection between socio-economic environment and self-concept is strengthened, and the need to address socio-economic disparities in the educational field is highlighted.

Despite socio-economic status acting as a moderating factor, it is recommended to develop educational initiatives that foster optimal self-concept development. According to Hellison [60], dimensions of perceived family involvement are differentially related to self-concept dimensions. Implementing workshops and lectures with the participation of the educational community is suggested, aiming to enhance self-concept from a psychological health perspective. Similar to the suggestions of Hellison [60], educational programs within the school context have been designed to maintain or enhance self-concept. These activities are based on the theories proposed by Álvarez et al. [61], where self-enhancement theorists advocate for interventions aimed directly at enhancing self-concept, for example, by providing students with affection and support through exercises that generally stimulate and strengthen self-feelings. Conversely, skill development theorists argue that self-attitudes are the outcomes of academic achievement, and thus, educational efforts should focus on achieving specific academic skills. In recent years, the emphasis has shifted towards skill-learning interventions directed at specific dimensions [62].

## 5. Conclusions

The previous section analyzed the results obtained in detail, and this section will present the conclusions related to the objectives and hypotheses formulated. This section delves into the central problem of the research, which focuses on raising awareness about the issue of mental health in primary education students. In relation to the first specific objective of this study, which sought to analyze the connection between parental socialization styles and self-concept in the field of physical education, it has been observed that parental styles do have an impact on the self-concept of primary education students. This confirms the hypothesis proposed, which holds that the parental style received by primary education students conditions the development of their self-concept. Marín [59] emphasizes that the area of physical education considers values as fundamental in the teaching–learning process. Hellison [60] proposed a pedagogical model of personal and social responsibility, in which the importance of values such as overcoming, autonomy, cooperation and responsibility in physical education is emphasized. These values promote interaction and relationships with others. Consequently, it is possible to find a balance between the values promoted in school and those transmitted through parental styles, which contribute to the formation of the self-concept of primary education students. In summary, this study has demonstrated that parental socialization styles have an impact on the self-concept of primary education students in the field of physical education. These findings support the importance of considering the values promoted both in the school environment and at home, as both play a significant role in shaping the self-concept of students. It has been concluded that the democratic parental socialization style has a negative effect on the perception of self-esteem, while the authoritarian parental style modulates the perception of physical appearance, physical condition and strength. Additionally, it was observed that the negligent parental style affects the development of perceived competence. On the other hand, it was found that the permissive parental socialization style has the strongest connection with the development of all subcategories of self-concept. In the case of the permissive parental style, it is noted that it grants greater freedom to the child in their day-to-day life, allowing them to correct their own mistakes and face new experiences without direct influence from the family environment. In relation to self-concept as a consequence of parental socialization styles, it is concluded that this factor is decisive for students to start in sports and develop in the best possible conditions. Having a high level of self-concept, children will have greater self-esteem and confidence in themselves to interact with others and face both educational and sports challenges. Regarding the second objective, the findings of this study offer important contributions to the field of developmental psychology. The significant association between socio-economic environment and self-concept underscores the importance of considering social and economic contexts in understanding identity formation in adolescence. Additionally, the results suggest that factors other than parental styles might play an important role in the development of self-concept at this life stage. In conclusion, this study provides evidence that the socio-economic environment has a significant relationship with the self-concept of adolescents. Although no significant difference was found in relation to parental styles, further research is needed that considers additional factors and employs more comprehensive methodological approaches. Better understanding the complex interactions between socio-economic environment, parental styles and self-concept is crucial for promoting the healthy development of adolescents and designing appropriate interventions in this field. Finally, regarding the third objective, these findings have important implications for education and pedagogical practice. By recognizing the connection between the socio-economic environment and students’ self-concept, education professionals can design strategies and programs that promote healthy self-concept development in all students, regardless of their socio-economic background. Additionally, these results highlight the importance of addressing socio-economic inequalities in the educational field, with the aim of creating inclusive environments that foster the comprehensive development of students.

## 6. Limitations

Regarding the limitations identified in this research study, a series of factors were recognized that could have influenced its development and outcomes, and measures were implemented to address these limitations. It is important to note that in social science research, it is common to encounter certain restrictions that cannot be fully controlled, as indicated by Baños et al. [63]. Regarding the instruments used in the research, quantitative questionnaires were employed, and the absence of a qualitative methodology is acknowledged. According to Sánchez et al. [64], combining both methodologies in research can strengthen it and provide solidity in interpreting the results as the convergence of findings obtained through both methodologies increases the validity of the results. However, the length of the questionnaires used in this study was a limitation for the participation of educational centers. Specifically, it was found that the PSPP questionnaire was extensive and difficult for primary education students to understand. Díaz [65] suggests that lengthy questionnaires with complex questions can hinder the collection of precise information, increase the risk of non-response and make it difficult to obtain a high rate of complete questionnaires. Most questionnaires were delivered and supervised by teachers, which represented a limitation as it was not possible to be present to resolve doubts. To ensure coherent responses, explanations of the questionnaires were provided to those in charge of administering them. During the administration process, it was considered that the use of four questionnaires could have contributed to non-response due to the mental fatigue that can arise from completing several questionnaires in succession. Additionally, the participation of educational centers was limited due to the COVID-19 pandemic. It is mentioned that the quantitative approach used in the questionnaires hindered the precision of the study, which could be improved with the incorporation of qualitative techniques, such as interviews with education professionals related to the topic. Bevins [25] noted that the non-response effect can affect the coherence of responses in questionnaires. On the other hand, Bevins [25] highlighted that the ex post facto design presents advantages and disadvantages. Among the advantages, its usefulness for analyzing cause and effect in the study object is highlighted, and it is more economical and requires less working time than an experimental investigation. However, among the disadvantages is that the researcher cannot manipulate the study variables nor clearly establish a relationship between the independent and dependent variables being examined.

## 7. Practical Implications

This section presents the educational implications derived from the results obtained in this research conducted with primary education students. Although socio-economic level was identified as a factor which affects the issue, it is suggested to implement initiatives in the educational context to promote healthy eating. Among these initiatives are workshops and conferences involving students, their families and the rest of the school’s teaching staff. The goal is to encourage reflection and critical thinking about the importance of having optimal self-concept development. In summary, the idea that youth should not be considered simply as a reduced version of adults but as a stage of formation for effective social integration is defended. In this process, both the educational community and the family play a fundamental role. Therefore, the need to work jointly among all involved parties to achieve complete and appropriate training for young people is proposed. This idea is supported by the global view of society, which recognizes the importance and relevance of the youth stage in the process of formation and social development.

## Figures and Tables

**Table 1:** Sociodemographic characteristics of the participant sample.

Variable	Category	n (%)
Sex	Men	467 (49.84%)
	Women	470 (50.16%)
Age	9	26 (2.77%)
	10	48 (5.12%)
	11	378 (40.35%)
	12	431 (46.01%)
	13	53 (5.65%)
	14	1 (0.10%)
Course	4°	47 (5.02%)
	5°	399 (42.58%)
	6°	491 (52.40%)
Type of center	Public	507 (54.10%)
	Subsidized/Private	430 (45.90%)

**Table 2:** Relationship between parental socialization styles and self-concept.

Self-Concept	Total	Parental Style				*p*
		Democratic	Authoritarian	Permissive	Negligent	
Physical appearance	2.80 ± 0.65	2.86 ± 0.62	2.62 ± 0.66	2.95 ± 0.62	2.77 ± 0.65	0.000
Physical condition	2.96 ± 0.63	3.04 ± 0.62	2.78 ± 0.63	3.19 ± 0.61	2.86 ± 0.65	0.000
Perceived competence	2.85 ± 0.80	2.90 ± 0.78	2.75 ± 0.80	3.05 ± 0.81	2.72 ± 0.76	0.000
Strength	2.69 ± 0.67	2.74 ± 0.64	2.61 ± 0.66	2.80 ± 0.73	2.63 ± 0.64	0.011
Self-esteem	2.92 ± 0.71	2.75 ± 0.70	2.81 ± 0.62	3.09 ± 0.69	2.87 ± 0.71	0.001
Total (N)	783	210	190	174	209	

**Table 3:** Relationship of parental socialization styles and socio-economic environment with self-concept.

Self-Concept	Total	Parental Style				*p*
		Democratic	Authoritarian	Permissive	Negligent	
Physical appearance	2.80 ± 0.65	2.86 ± 0.62	2.62 ± 0.66	2.95 ± 0.62	2.77 ± 0.65	0.000
Physical condition	2.96 ± 0.63	3.04 ± 0.62	2.78 ± 0.63	3.19 ± 0.61	2.86 ± 0.60	0.000
Perceived competence	2.85 ± 0.80	2.90 ± 0.78	2.75 ± 0.80	3.05 ± 0.81	2.72 ± 0.76	0.000
Strength	2.69 ± 0.67	2.74 ± 0.64	2.61 ± 0.66	2.80 ± 0.73	2.63 ± 0.64	0.011
Self-esteem	2.92 ± 0.71	2.75 ± 0.70	2.81 ± 0.62	3.09 ± 0.69	2.87 ± 0.71	0.001
Total (N)	783	210	190	174	209	

**Table 4:** Relationship between family socio-economic environment and self-concept.

Self-Concept	Family Socio-Economic Level (*p*)
Physical appearance	0.037
Physical condition	0.001
Perceived competence	0.008
Strength	0.116
Self-esteem	0.001

**Table 5:** Association between mother’s socio-economic level and self-concept.

Self-Concept	Mother’s Socio-Economic Level
	(*p*)
Physical appearance	0.021
Physical condition	0.006
Perceived competence	0.128
Strength	0.038
Self-esteem	0.001

**Table 6:** Association between father’s socio-economic level and self-concept.

Self-Concept	Father’s Socio-Economic Level
	(*p*)
Physical appearance	0.083
Physical condition	0.003
Perceived competence	0.009
Strength	0.555
Self-esteem	0.001

## Data Availability

Data are contained within the article.
